# A Zinc–Bromine Battery with Deep Eutectic Electrolytes

**DOI:** 10.1002/advs.202204908

**Published:** 2022-10-30

**Authors:** Jiyun Heo, Kyungjae Shin, Hee‐Tak Kim

**Affiliations:** ^1^ Department of Chemical and Biomolecular Engineering Korea Advanced Institute of Science and Technology 291, Daehak‐ro Yuseong‐gu Daejeon 34141 Republic of Korea; ^2^ Advanced Battery Center KAIST Institute for the NanoCentury KAIST 291, Daehak‐ro Yuseong‐gu Daejeon 34141 Republic of Korea

**Keywords:** anode‐less system, deep eutectic electrolytes, flexible pouch cell, halogen chemistry, Zn–Br batteries

## Abstract

A deep eutectic solvent (DES) is an ionic liquid‐analog electrolyte, newly emerging due to its low cost, easy preparation, and tunable properties. Herein, a zinc–bromine battery (ZBB) with a Zn‐halide‐based DES electrolyte prepared by mixing ZnBr_2_, ZnCl_2_, and a bromine‐capturing agent is reported. The water‐free DES electrolyte allows a closed‐cell configuration for the ZBB owing to the prevention of Br_2_ evaporation and H_2_ evolution. It is found that the Cl^−^ anion changes the structure of the zinc‐halide complex anions and demonstrated that it improves the ion mobility and electrode reaction kinetics. The DES electrolyte with the optimized ZnCl_2_ composition shows much higher rate capability and a cycle life 90 times longer than that of a ZnCl_2_‐free DES electrolyte. A pouch‐type flexible ZBB battery based on the DES electrolyte exhibits swelling‐free operation for more than 120 cycles and stable operation under a folding test, suggesting its potential in consumer applications such as wearable electronics.

## Graphical abstract

Deep eutectic electrolytes are specifically designed for use in zinc–bromine batteries (ZBBs). By virtue of the water‐free property and superb Br_2_ adsorption ability of these electrolytes, problematic gas‐related issues are effectively suppressed, allowing the operation of ZBBs in consumer‐friendly configurations. The essential role of Cl^−^ with regard to improving the performance capabilities of ZBBs has also been thoroughly studied.

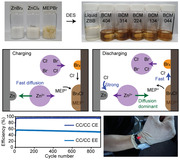

## 1 Introduction

Cost‐effective new battery systems are consistently being developed to meet a range of energy demands. Zinc–bromine batteries (ZBBs) are considered to represent a promising next‐generation battery technology due to their low cost, high energy densities, and given the abundance of the constituent materials.^[^ ^1^ ^]^ The positive electrode reaction of a ZBB based on the Br_2_/Br^−^ redox couple is attractive because it shows a high redox potential (1.07 V vs a standard hydrogen electrode (SHE)) and a high specific capacity (335 mAh g^−1^).^[^ ^2^ ^]^ Moreover, the Zn^2+^/Zn redox couple has garnered much interest over the last few decades as the negative electrode material considering its high specific capacity (820 mAh g^−1^) and decent redox potential (−0.76 V vs SHE).^[^ ^3^ ^]^ Owing to these advantages, ZBBs feature high practical energy density (70 Wh kg^−1^) and a high discharge voltage (1.83 V).^[^ ^4^ ^]^ Another strong motivation to use the ZBB chemistry is the low cost of the active material, ZnBr_2_. The energy cost of ZnBr_2_ is only 5–8 $ kWh^−1^, among the lowest reported thus far and six times lower than that of lithium ion batteries, demonstrating the economic advantage of ZBBs for consumer applications.^[^ ^5^, ^6^ ^]^

However, ZBBs suffer from severe gas generation, which deteriorates the electrochemical cycling performance and limits ZBB designs given the expensive electrolyte‐flowing system required. One relevant issue is the evaporation of the Br_2_ that forms via the oxidation of Br^−^ at the positive electrode during the charging process (Equation (1))^[^ ^7^ ^]^

(1) $$2{\mathrm{Br}}^{-}\to {\mathrm{Br}}_{2}↑+2e^{-}$$

Considering that Br_2_ molecules in the gas phase cannot participate in the electrode reaction, Br_2_ evaporation results in a loss of charged energy and a decrease of the coulombic efficiency (CE).^[^ ^8^ ^]^ In addition, special care should be taken to prevent the leakage of Br_2_ gas due to the corrosiveness and toxicity of Br_2_. The conventional means of alleviating Br_2_ evaporation is to use a Br‐capturing agent (BCA) as an electrolyte additive.^[^ ^9^ ^]^ The BCA captures Br_2_ by forming a complex of BCA and Br_2_, lowering the vapor pressure of Br_2_. However, there is a practical limitation to Br_2_ capturing for conventional aqueous ZBBs because charged Br species do dissolve in water, slowly releasing Br_2_ gas by vaporization. Hydrogen evolution is another detrimental reaction that reduces the CE of a ZBB.^[^ ^10^ ^]^ H_2_ is highly prone to arise at the negative electrode in ZBBs during the charging process owing to the acidic electrolytes (pH < 4) used for ZBBs.^[^ ^11^ ^]^ Accumulation of these gases in the cell is prevented by an electrolyte flowing system for practical ZBBs. For this reason, the importance of minimizing the gas generation reaction is seldom addressed. However, in order to endow flexibility in the cell design for ZBBs and achieve wide acceptance of the cost‐effective Zn–Br chemistry in consumer applications such as the pouch format, new strategies for preventing gas generation must be developed.

A deep eutectic solvent (DES) is a eutectic mixture of Lewis or Brønsted acids and bases.^[^ ^12^ ^]^ Due to its simplicity in preparation, low cost, low‐toxicity, and tunable chemical property, it is widely used in various fields including separation, synthesis, and electrochemical process.^[^ ^13^, ^14^ ^]^ A DES can be also used as an electrolyte for batteries as exemplified by the DES‐based lithium battery and zinc‐organic battery.^[^ ^15^, ^16^ ^]^ The ionic liquid‐like ion conducting property of a DES renders it a possible electrolyte material option; however, DES‐based battery technology is still in its infancy. With regard to the possible use of DESs in battery applications, we paid attention to halide‐based DESs for ZBBs considering their potential to provide Br^−^ conduction, the high solubility of Br_2_, and the Br_2_ capturing ability.^[^ ^17^ ^]^

In this work, we report a novel ZBB with DES electrolytes that fundamentally addresses the Br_2_ evaporation and H_2_ evolution issues. The DES electrolyte is prepared by mixing a quaternary ammonium salt, zinc bromide (ZB), and zinc chloride (ZC). Quaternary ammonium salts, which are the most widely used BCAs, stabilize Br_2_ by forming complexes, and the water‐free feature of the DES electrolyte ensures that H_2_ evolution does not occur. We discovered that a critical role of Cl^−^ anions is to increase the ion mobility of the DES electrolyte and facilitate positive and negative electrode reactions. For a DES with the optimal Cl^−^ content, we could operate an anode‐less ZBB in closed coin‐cell configuration for more than 900 cycles. Addressing the two critical problems also allowed the design of a pouch‐type flexible ZBB, and the successful operation of the flexible DES‐ZBB upon folding and unfolding is presented. This first ever demonstration of a DES‐based ZBB will herald greater flexibility in the design of metal‐halide‐based batteries.

## 2 Results and Discussion

### 2.1 Development of DESs for ZBBs

When designing the DES as a ZBB electrolyte, the following five points were considered: (1) the DES should be in a liquid state at room temperature, (2) salts containing Zn^2+^ and Br^−^ ions must be included in the DES to enable both Zn^2+^/Zn and Br_2_/Br^−^ redox reactions, (3) an effective BCA must be included to suppress Br_2_ evaporation, (4) DES should have high ionic conductivity, and (5) should be compatible with the electrode reactions of ZBBs. First, we investigate the formation of the DES for the three widely used BCAs (1‐ethyl‐1‐methylpyrrolidinium bromide (MEPBr), tetrapropylammonium bromide (TPABr), and tetrabutylammonium bromide (TBABr)) in combination with ZnBr_2_ (ZB). As shown in Figure S1 (Supporting Information), only MEPBr resulted in a liquid‐phase DES at room temperature, which led to a focused investigation of the MEPBr‐containing DES in this work.

**Figure 1:**
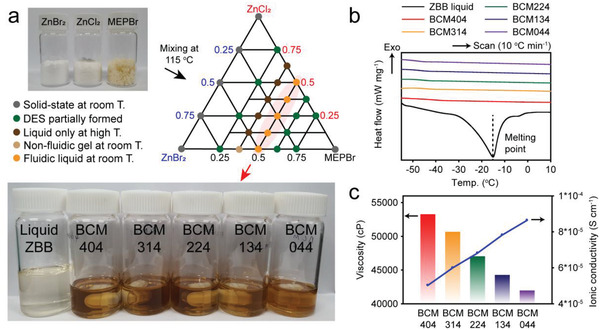
Characteristics of DESs based on zinc bromide (ZB), zinc chloride (ZC), and MEPBr. a) ZB‐ZC‐MEPBr ternary diagram for DES preparation. The top inset image shows the solid‐state parent salts. The bottom inset image shows the liquid‐state DES electrolytes together with a conventional aqueous ZBB electrolyte (2 m ZB + 0.5 m MEPBr). b) Differential scanning calorimetry analysis of the aqueous ZBB electrolyte and the DESs (BCM404, 314, 224, 134, and 044). c) Comparison of the viscosity and ionic conductivity of the DESs.

DESs based on ZB and MEPBr satisfies the first three aforementioned requirements. However, it is not capable of providing high ionic conductivity and a fast Zn redox reaction, which will be presented in the following section. To boost the electrochemical processes, we adopted ZC as an additional constituent component because Cl^−^ is known to increase the performance of the Zn anode reaction by complexing with Zn ions.^[^ ^18^ ^]^ The DESs studied in this work are denoted as BCMXYZ, where X, Y, and Z are the molar ratios of ZB, ZC, and MEPBr, respectively. As shown in the ternary diagram in **Figure** **1**a, the phase of the BCM DES varies precisely depending on the molar composition. The optical images of the BCM mixtures studied in this work are provided in Figure 1a and Figure S2 (Supporting Information). A homogeneous and transparent mixture was obtained in a narrow range of (ZB+ZC)/MEPBr ratios between 1.67/1 and 1/1, with the Zn halide salts remaining as a solid powder at the other compositions. To ensure high ionic conductivity, the DES should be in fluidic liquid state, and this requirement is fulfilled only when the (ZB+ZC)/MEPBr ratio is close to 1/1, as shown in the optical images of the typical DESs here, in this case BCM404, BCM314, BCM224, BCM134, and BCM044 (Figure 1a). The five liquid‐state DESs did not show any melting transition above − 55 °C, as observed in their differential scanning calorimeter thermograms (Figure 1b), in contrast to the melting transition (− 15 °C) of the conventional ZBB electrolyte (2 m ZnBr_2_ + 0.5 m MEMBr).

For the liquid‐state DESs with a (ZB+ZC)/MEPBr ratio of 1, the viscosity and ionic conductivity properties were investigated. As shown in Figure 1c, the viscosity gradually decreased and the ionic conductivity increased with an increase in the ZC content, indicating a positive effect of Cl^−^ on the ion transport. Although a similar impact of the addition of Cl^−^ was reported in aqueous ZBBs, the fundamental reason for the enhancement in the DES electrolytes here differs from that in aqueous electrolytes.^[^ ^19^ ^]^

**Figure 2:**
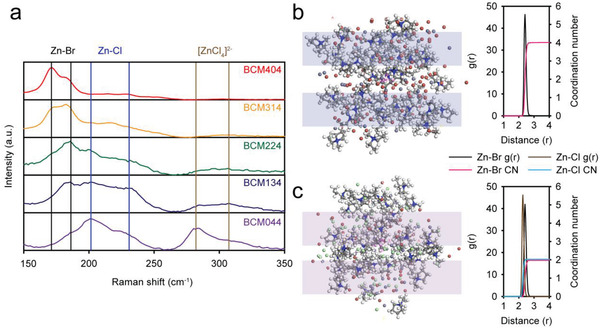
Structure of BCMs with different ZB/ZC ratios. a) Raman spectra of BCM 404, 314, 224, 134, and 044. MD simulation results for b) BCM404 and c) BCM134. The figures on the right in (b) and (c) are the RDF results. Those on the left in (b) and (c) are snapshots of the MD simulation results. Atom colors in (b) and (c) are as follows: H‐white, C‐gray, N‐blue, Zn‐dark gray, Br‐brown, and Cl‐cyan. Colored boxes indicate MEP^+^ cation layers separated by Zn‐halide anion complex layers.

To understand the effect of Cl^−^ on the structure of the DES, we analyzed the structure of the BCM DESs using Raman spectroscopy, ^13^C solid‐state NMR spectroscopy, density functional theory (DFT) calculations, and a molecular dynamics (MD) simulation. The Raman spectra of the BCMs with different ZB/ZC ratios are shown in **Figure** **2**a. With an increase in the ZC content in the BCMs, the peaks from [ZnCl*_x_*]^(2−^ *^x^* ^)^ (200–230 cm^−1^) and the deformation vibration peak from [ZnCl_4_]^2−^ (280–320 cm^−1^) became more pronounced, while the peaks related to the [ZnBr*_x_*]^(2−^ *^x^* ^)^ (170–190 cm^−1^) were diminished.^[^ ^20^, ^21^, ^22^, ^23^ ^]^ Moreover, peaks corresponding to the Zn–Br bond were observed even for BCM044 (Figure S3, Supporting Information), which does not include ZB. This indicates that the MEPBr undergoes a reconstruction process, resulting in a phase change from a solid to an ionic liquid‐like state due to the complexation of Br^−^ in the MEPBr and the Zn^2+^ in the other components.

From the DFT calculations, the most stable conformation of the powder MEPBr is obtained when Br^−^ closely interacts with the methyl group in the MEP^+^ (Figure S4a, Supporting Information). However, for the DESs, the counter anion ([ZnBr_4_]^2−^) is stabilized when it is adjacent to the ethyl group in the MEP^+^ (Figure S4b, Supporting Information). This type of reconstruction process of the MEPBr is experimentally validated by the Raman and solid‐state ^13^C NMR spectra shown in Figure S5 (Supporting Information). According to the shifts in the Raman spectra, the bond related to the methyl group shrank while those of ethyl group stretched as the DESs were formed.^[^ ^24^, ^25^, ^26^, ^27^ ^]^ Similarly, the DES electrolyte showed a down‐field shift (deshielding) of the methyl group peaks, whereas an up‐field shift (shielding) was noted for the peaks related to the ethyl group compared to the powder MEPBr, depicting the stabilization process observed in the DFT results.^[^ ^28^ ^]^ These results, therefore, indicate that the conformation of MEPBr readily changes to allow Br^−^ to form a Zn‐halide complex anion with Zn halide salts. The redistribution of Br^−^ in the DES is quite feasible considering the much greater binding energy between Br^−^ and Zn^2+^ (− 14.1 eV) in comparison with that between MEP^+^ and Br^−^ (− 4.2 eV).

To understand the molecular arrangement in the DES, a MD simulation was carried out with two typical DESs: BCM404 and BCM134. Figure 2b,c presents snapshots of the simulation result, the radial distribution function (RDF), and the coordination number for BCM404 and BCM134, respectively. As displayed in the snapshot images, the BCM DESs form a liquid‐crystal‐like morphology featuring alternating layers of MEP cations (boxed regions in the images) and Zn‐halide complex anions. This phenomenon is in line with the local ordering of ions found for highly concentrated electrolytes (>10 m).^[^ ^29^ ^]^

The structure of the Zn‐halide complex anion was investigated further by means of RDF analysis. BCM404 exhibited coordination of the central Zn^2+^ cation with four Br^−^ ions with a distance of (2.41 Å). On the other hand, BCM134 showed coordination numbers of 2 for Br^−^ and 2 for Cl^−^ anion, indicating the formation of the [ZnBr*_x_* Cl_4−_ *_x_*]^2−^ complex. The Br^−^/Cl^−^ ratio of 2/2 in the complex is different from the stoichiometric ratio of ZB/ZC (1/3), which corroborates the extraction of Br^−^ from MEPBr during the formation of the complex.

Moreover, for BCM134 (Figure 2c), the distances of Zn–Cl and Zn–Br are different (2.27 and 2.41 Å, respectively), signifying that the size of the Zn‐halide complex anion is reduced with the involvement of Cl^−^. This outcome provides a clue with which to understand the positive effect of Cl^−^ on the ionic conductivity. The reduced size of the Zn‐halide complex can increase its mobility, resulting in an enhancement of the ionic conductivity of the DESs. Moreover, the reduced viscosity with an increase in the content of Cl^−^ anions can be explained by the weaker halogen bond strength of the Cl^−^ anion compared to that of the Br^−^ anion, which can reduce the mutual interaction among the complexes.^[^ ^30^ ^]^

### 2.2 Effect of Cl^−^ Anions on the Cycling Performance of the DES‐ZBB

BCM‐DES‐based hermetically sealed ZBBs were constructed using the coin‐type cell format and their electrochemical performances were investigated. Considering that the ionic species of BCM DESs differ from that of the conventional aqueous ZBB electrolyte, first we attempted to observe whether the DES‐based ZBB (DES‐ZBB) would also be capable of using the Zn^2+^/Zn and Br^−^/Br_2_ redox reaction for energy storage, as does the aqueous ZBB. As shown in Figure S6 (Supporting Information), Zn deposition at the negative electrode and the formation of bromine species at the positive electrode were identified after the first charge process using the BCM404 electrolyte.^[^ ^31^ ^]^ This is a clear sign of dual‐ion redox reactions, which resemble the charge process of the conventional ZBB, demonstrating the feasibility of the DES‐ZBB.

**Figure 3:**
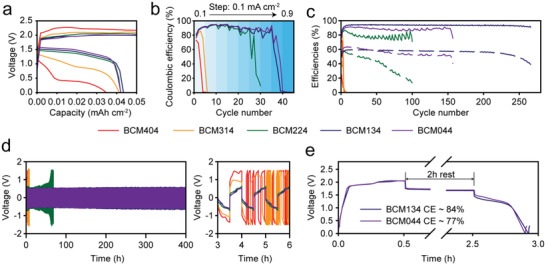
Electrochemical performances of DES‐ZBBs with different ZB/ZC ratios at 25 °C. a) voltage profiles of DES‐ZBBs, b) rate capability test of DES‐ZBBs with varying current densities from 0.1 to 0.9 mA cm^−2^ with a fixed areal capacity (0.05 mAh cm^−2^ charged), c) long‐term cycling stability test (solid line: coulombic efficiency, dash line: energy efficiency) of the DES‐ZBBs, and d) galvanostatic cycling test using a symmetric Zn/Zn cell with BCM DESs. The figure on the right in (d) provides the voltage profiles of the BCM DESs early during the cycling process. e) OCV holding test for the BCM134 and BCM044 cells. All tests were conducted under the 0.1 mA cm^−2^ / 0.05 mAh cm^−2^ test condition, except for the rate capability test in (b).

**Figure** **3**a shows the voltage profiles of the DES‐ZBBs with BCM404, 314, 224, 134, and 044. The charge and discharge overpotentials decreased remarkably with an increase in the ZC/ZB ratio (the detailed explanation of the role of Cl^−^ on the overpotential is discussed in the next section and Note S1, Supporting Information). Due to the large discharge overpotential, ZC‐free BCM404 delivered the lowest discharge capacity. The rate capability performance of the BCM DESs also showed strong dependency on the ZB/ZC ratio. As shown in Figure 3b, the BCM404 and BCM314 cells failed at 0.1 mA cm^−2^, whereas BCM224 operated up to 0.5 mA cm^−2^ and the BCM DESs with a higher Cl^−^ content (BCM134 and BCM044) operated even under 0.7 mA cm^−2^. The strong dependency of the overpotential and rate capability on the ZC content signifies the essential role of the Cl^−^ anion with regard to boosting the redox reactions.

The long‐term cycling performance was examined for the BCM DESs, as shown in Figure 3c. The cycling stability of ZBBs is generally enhanced with the increment of the ZC content. BCM404 and BCM314 showed fast failure in early cycles. BCM224 exhibited a longer cycle life than BCM404 and BCM314, demonstrating enhanced cycling stability due to the addition of ZC. The positive effect of ZC was more pronounced for BCM134 and BCM044, and only these electrolytes showed decent cycling performance over 100 cycles. However, BCM134 delivered longer cycling stability and higher coulombic efficiency than BCM044. This indicates that there is an optimum ZC content that effectively prolongs the cycle life of DES‐ZBBs.

The cycling stability of the ZBBs appears to be determined by the reversibility of the Zn electrode. As shown in Figure 3d, the cycling stabilities of the Zn/Zn symmetric cells are in good agreement with those observed for the ZBBs. Due to the large overvoltage, the Zn plating/stripping capacity became smaller in the early cycles for BCM404 and BCM314. In contrast, BCM134 and BCM044 exhibited outstanding reversibility of the Zn plating/stripping reaction (Figure S7, Supporting Information), which confirms that Cl^−^ is advantageous for reducing the overvoltage and enhancing the reversibility of the Zn electrode reaction.

In the comparison of BCM134 and BCM044, BCM134 showed higher cycling stability and higher coulombic efficiency than BCM044 despite its lower ZC content. BCM134 showed the most stable voltage profile during the cycling test (Figure S8, Supporting Information) and operated stably for 270 cycles with the highest CE (≈93%) amongst the BCMs. This behavior can be considered in terms of self‐discharge resistibility. For BCM134 and BCM044, self‐discharge rates were compared by storing the charged cells for 2 h at the OCV and measuring the subsequent discharge capacity (Figure 3e); the CE was 84% for BCM134 and 77% for BCM044 in the OCV holding test, indicating that an excess amount of Cl^−^ can accelerate not only the transport of ions but also the cross‐over of charged Br species because it reduces the viscosity of the electrolyte medium.

Additional tests were also conducted using BCM134 electrolyte in order to observe the capacity capability of DES‐ZBB. The symmetric cell stably operated over the tested areal capacity range (0.05–0.25 mAh cm^−2^) as shown in Figure S9 (Supporting Information). On the other hand, for the Zn/CC full cell, performance fade was observed at areal capacities above 0.2 mAh cm^−2^, probably due to the prolonged self‐discharge reaction. In addition to the stable cell cycling at 0.05 mAh cm^−2^ shown in Figure 3c, we could further demonstrate a stable operation of the DES‐ZBB at 0.1 mAh cm^−2^. It should be also noted that ZBB operation in a closed configuration such as a coin‐type cell is hardly achievable with a conventional aqueous electrolyte due to the evolution of H_2_ and the evaporation of Br_2_.^[^ ^32^ ^]^ However, the DES‐ZBB did not exhibit any cell swelling or venting during the extended cycling test (Figure S10, Supporting Information). This is due to the absence of H_2_O (Figure S11, Supporting Information) and the low vapor pressure of Br_2_ in the BCMs (Figure S12, Supporting Information), convincingly demonstrating the effectiveness of the DES electrolyte with regard to minimizing gas generation in the ZBB.

### 2.3 The Roles of Cl^−^ Anions in the Negative and Positive Electrode Reactions

**Figure 4:**
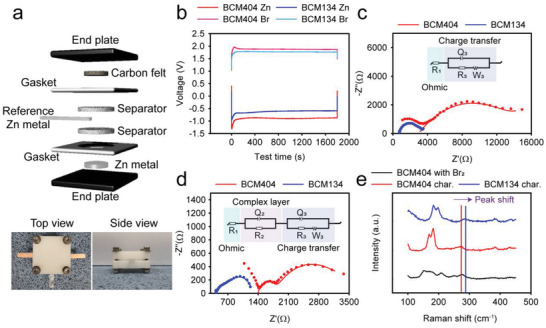
Electrochemical analyses of the three‐electrode BCM404 and BCM134 cells to elucidate the effect of Cl^−^. a) the custom‐made three‐electrode system used for the analyses, b) voltage profiles of the negative and positive electrodes when charging. The EIS spectra of c) the charged negative electrode and d) charged positive electrode (charging capacity: 0.05 mAh cm^−2^) are shown. e) Raman spectra of the charged BCM404 and BCM134 in comparison with the mixture of BCM404 and liquid Br_2_.

Although DES‐ZBB shares the same redox reaction on both the negative and positive electrode with the conventional liquid electrolyte‐based ZBB (Figure S6, Supporting Information), the performance‐limiting factor of the DES‐ZBB may differ from that of the conventional ZBB due to the different ionic structure of the reactant ions (Zn^2+^ and Br^−^). Moreover, the strong effect of Cl^−^ anions on the cell performances cannot simply be explained by the increment of the ionic conductivity with Cl^−^ anions. In relation to this, further investigations were conducted of the electrochemical reactions at the positive and negative electrodes for BCM404 and BCM134 using a three‐electrode system, as shown in **Figure** **4**a.

Figure 4b shows the electrode potential profiles (versus a Zn pseudo reference electrode) for the BCM404 and BCM134 ZBBs when charging under the 0.1 mA cm^−2^/0.05 mAh cm^−2^ condition. The upper potential profiles correspond to the Br^−^ oxidation reaction at the positive electrode, and the lower potential profiles correspond to the Zn^2+^ reduction reaction at the negative electrode. The nominal Br^−^ oxidation potentials are 1.88 and 1.77 V for BCM404 and BCM134, respectively. Considering the thermodynamic Br_2_/Br^−^ redox potential for BCM404 and BCM134 (Table S1; 1.74 and 1.76 V, respectively, Supporting Information), the overpotential of the Br^−^ oxidation reaction is much lower for BCM134 (0.01 V) than it is for BCM404 (0.14 V). Moreover, the overpotentials of the Zn^2+^ reduction reaction were measured and found to be − 0.88 and − 0.61 V for BCM404 and BCM134, respectively, verifying a lower overpotential for BCM134 than BCM404. We also conducted a cyclic voltammetry (CV) test using the three‐electrode cell (Figure S13, Supporting Information). Both BCM404 and BCM134 exhibited characteristic CV curves of Zn/Br redox reactions, however, BCM134 showed higher current densities for the Zn and Br redox reactions. These results confirm that the addition of ZC can boost both positive and negative electrode reactions.

The three‐electrode cell test also demonstrated that the overvoltage values of the DES‐ZBBs are dominated by the negative electrode reaction (Zn^2+^/Zn reaction), in contrast to the conventional ZBBs based on an aqueous electrolyte in which the Zn^2+^/Zn reaction is faster than the Br_2_/Br^−^ redox reaction.^[^ ^33^, ^34^, ^35^ ^]^ The comparison of the migration behavior of Zn^2+^ between the two electrolytes provides a clue pertaining to the sluggish Zn^2+^/Zn reaction with the DES electrolyte. For an aqueous electrolyte, a form of hydrated Zn^2+^, [Zn(H_2_O)_6_]^2+^, is supplied to the negative electrode during the charging process and is removed from the negative electrode when discharging via migration, facilitating the Zn^2+^/Zn redox reaction. However, in the DES electrolytes, Zn^2+^ migrates in the opposite direction due to the formation of the [ZnBr*_x_* Cl_4−_ *_x_*]^2−^ complex anion. This obstructs Zn^2+^/Zn reaction because the supply and removal of Zn^2+^ are hindered by the migration. Thus, the diffusion of [ZnBr*_x_* Cl_4−_ *_x_*]^2−^ governs the rate of the Zn^2+^/Zn reaction, causing a large overpotential at the negative electrode.

To examine more closely the effects of ZC, an EIS analysis was conducted using the three‐electrode configuration. Figure 4c shows Nyquist plots of the negative electrodes for BCM404 and BCM134 after charging for 30 min at 0.1 mA cm^−2^. For a qualitative comparison, the equivalent circuit model shown in Figure 4c was fit to the impedance data and the *R*_ct_ values were determined, as provided in Table S2 (Supporting Information). This comparison found that the inclusion of the Cl^−^ anion decreases not only the electrolyte resistance (ohmic resistance) but also the charge transfer resistance. As mentioned above, the negative electrode reaction in the DES‐ZBB strongly depends on the diffusion process of the Zn‐halide complex anion. Because the inclusion of the Cl^−^ anion in the complex decreases the size of the complex (Figure 2), the supply of Zn^2+^ to the electrode surface via the diffusion process can be faster for BCM134 compared to BCM404, resulting in a faster redox reaction. Another possible advantageous effect is the acceleration of the Zn oxidation process by the strong interaction between Cl^−^ and Zn^2+^.^[^ ^18^ ^]^ As indicated by our DFT calculations, Cl^−^ (− 15.5 eV) undergoes stronger interaction than Br^−^ (− 14.1 eV) with Zn^2+^ and can therefore more effectively stabilize the Zn^2+^ ion by rapidly adsorbing Zn^2+^ into the DES and thus promote the oxidation of Zn to Zn^2+^.

Nyquist plots of the impedances of the positive electrode reaction for BCM134 and BCM404 are also compared in Figure 4d. The higher frequency semicircle is attributable to ion migration through the complex layer of the MEP cation and the polyhalogen anion complex, and the lower frequency semicircle stems from the charge transfer process coupled with the diffusion of the redox species.^[^ ^36^ ^]^ Quantification of the charge transfer resistance was conducted by curve fitting using the circuit model shown in Figure 4d. In accord with the smaller positive electrode overpotential for BCM134 (Figure 4b), the charge transfer resistance for the Br_2_/Br^−^ redox reaction was smaller for BCM134 (Table S2, Supporting Information). While the lower charge transfer resistance of BCM134 can be understood by considering the enhanced diffusion process, we noted an additional effect of Cl^−^ on the Br_2_/Br^−^ redox reaction kinetics from the Raman spectra of the charged BCMs. As shown in Figure 4e, the electrochemically charged BCM404 and chemically charged BCM404 (with liquid Br_2_ added to BCM404) showed characteristic peaks of Br^5−^ (reportedly the most predominant species of charged polybromides) at 275 cm^−1^, indicating that charged bromine is stabilized in the form of a polybromide anion species (e.g., Br^3−^, Br^5−^).^[^ ^37^ ^]^ However, the charged BCM134 showed a peak shift toward a higher wavenumber (290 cm^−1^) with respect to the BCM404 spectra. This phenomenon suggests that polyinterhalogen anions such as Br_2_Cl^−^ and Br_4_Cl^−^ form when Cl^−^ is included in the DES.^[^ ^38^, ^39^ ^]^

The inclusion of Cl^−^ decreases the stability of the polyinterhalogen anion owing to its small size and disruption of the symmetry of the anion.^[^ ^40^ ^]^ This is demonstrated by the different dissociation constants for the polybromide anion (*K*) and the polyinterhalogen anion (*K*_1_), which are defined in the following equilibrium equations

(2) $$\frac{{\left[{\mathrm{Br}}_{3}\right]}^{-}}{\left[{\mathrm{Br}}_{2}\right]\times {\left[\mathrm{Br}\right]}^{-}}=K$$

(3) $$\frac{{\left[{\mathrm{Br}}_{2}\mathrm{Cl}\right]}^{-}}{\left[{\mathrm{Br}}_{2}\right]\times {\left[\mathrm{Cl}\right]}^{-}}=K_{1}$$

The equilibrium constant for the dissociation and association of Br^3−^ (*K*, 16) is much higher than that of Br_2_Cl^−^ (*K*_1_, 1.14), implying that the formation of the polyinterhalogen anion increases the redox kinetics of bromine because the dissociation of polybromides, which is a prerequisite for the bromine reduction reaction, is facilitated.^[^ ^41^, ^42^ ^]^

**Figure 5:**
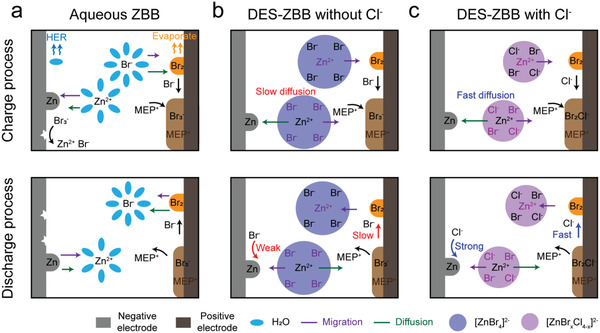
Schematic illustration of the charge (top) and discharge (bottom) processes of a) the aqueous ZBB, b) the DES‐ZBB without Cl^−^, and c) the DES‐ZBB with Cl^−^.

The effects of Cl^−^ on the ZBB performances are summarized in **Figure** **5**. This figure presents a comparison of the charge/discharge processes between the aqueous ZBB and the DES‐ZBB without/with the Cl^−^ anion. The conventional ZBB suffers from H_2_ evolution and Br_2_ evaporation (Figure 5a). In contrast, the DES‐ZBBs fundamentally eliminate H_2_ evolution at the negative electrode (Figure S11, Supporting Information) and effectively suppress Br_2_ evaporation by virtue of the sufficient amount of BCA and the low vapor pressure of Br_2_ in the DES (Figure S12, Supporting Information). These characteristics allow DES‐ZBBs to operate in closed cell configurations. Moreover, the inclusion of the Cl^−^ anion changed the properties of the BCMs considerably. Cl^−^ enhances the ionic conduction and diffusion processes by reducing the size of the Zn‐halide complex anion (Figure 5b,c). With the addition of Cl^−^, the Zn^2+^/Zn reaction is boosted by the faster diffusion of the smaller Zn‐halide complex anion and the stabilization of Zn^2+^ by Cl^−^. Furthermore, the formation of polyinterhalide anions in the presence of Cl^−^ accelerates the bromine reduction reaction (Figure 5c).

### 2.4 Flexible Pouch Type DES‐ZBB

The anode‐less ZBB, without a metallic Zn anode when initially assembled, presents a significant gain in the specific energy density and a cost reduction.^[^ ^43^ ^]^ The Zn deposit formed on the negative current collector upon the initial charge is the only source of the subsequent Zn^2+^/Zn reaction. Due to the zero‐excess use of the Zn source, strict prohibition of side reactions, including H_2_ evolution, is required to ensure high reversibility.^[^ ^44^ ^]^ Exploiting the H_2_ evolution‐free characteristic of the DES‐ZBB, an anode‐less ZBB was demonstrated by employing a carbon cloth (CC) current collector.

**Figure 6:**
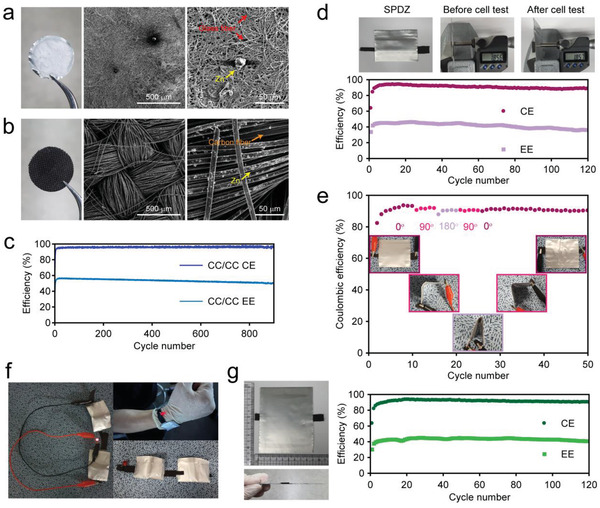
Demonstration of a flexible DES‐ZBB. Optical and SEM images of the negative electrodes taken out of a) the Zn||CC cell, and b) the CC||CC anode‐less cell after the first charge. c) Long‐term cycling stability test of the anode‐less coin‐type cell. d) (top) optical images of the as‐prepared SPDZ (active surface area of 1.5 cm^2^) and the measurement of the cell thicknesses before (1.86 cm) and after the long‐term cycling test (1.86 cm), and (bottom) the cycling performance of the SPDZ. e) Bend tolerance test at various bending angles (from 0° to 180°, and reversing back), f) LED emitting test of two series‐connected SPDZs, g) and optical image and cycling performance of a large‐area SPDZ (active surface area of 20 cm^2^). All tests were conducted under 0.1 mA cm^−2^ / 0.05 mAh cm^−2^ cycling condition.

**Figure** **6**a shows optical and SEM images of the negative electrode after the first charge process in Zn||CC (negative||positive) cells using the BCM134 electrolyte. In contrast to the pristine Zn metal electrode with the smooth surface morphology shown in Figure S14 (Supporting Information), the charged Zn metal electrode was covered with white glass fiber residue, which stemmed from the vertical growth of the Zn deposit and its entanglement with the adjacent glass fiber separator, a phenomenon frequently reported in Zn‐ion batteries.^[^ ^45^ ^]^ The inhomogeneous Zn deposition facilitates the formation of electrically isolated dead Zn during the discharge process and a self‐discharge reaction by expanding the contact with the charged Br species, impinging the reversibility of the Zn reaction. However, on the negative CC current collector of the anode‐less cell (CC||CC (negative||positive)), Zn deposits were uniformly spread over the CC surfaces, and the glass fiber residue was not observed (Figure 6b). Also, the size of the Zn deposits was significantly reduced compared to that on the Zn metal electrode. It has been reported that the surface oxygen‐containing functional groups in carbon materials provide the surface with high zicophilicity, resulting in a reduction of the Zn nucleation overpotential and well‐dispersed Zn nuclei over the carbon surface.^[^ ^46^, ^47^, ^48^, ^49^, ^50^ ^]^ The uniform Zn deposit morphology would be attributed to the oxygen‐containing surface defects (Figure S15, Supporting Information) and the large surface area of the 3D structured CC.

Because the Zn^2+^/Zn redox reaction dominates the performance of the DES‐ZBB (Figure 4), adopting a high‐surface‐area negative current collector improves the rate capability of the ZBB. While the Zn||CC cell failed to operate at 0.3 mA cm^−2^ and above, the CC||CC cell operated in the 0.3 mA cm^−2^ / 0.15 mAh cm^−2^ condition (Figure S16, Supporting Information). As shown in Figure 6c, the use of the CC electrode is also beneficial for enhancing the cycling stability. The cycle life of the DES‐ZBB extended from 270 cycles (Figure 3c, accumulated areal discharge capacity (AADC): 12.2 mAh cm^−2^) to 900 cycles (AADC: 42.4 mAh cm^−2^) when replacing the Zn foil electrode with the CC electrode.

Encouraged by the flexible characteristics of all components (CC electrode and glass fiber separator) of the anode‐less DES‐ZBB, we fabricated a soft package DES‐ZBB (SPDZ). It showed stable performance for more than 120 cycles even in the absence of stack pressure (Figure 6d). The thickness of the SPDZ was nearly invariant after a long‐term cycling test, which confirms the effective suppression of gas generation with the BCM DES. Also, the SPDZ exhibited stable performance upon a bending test (from a 0° (flat) to a 180° (completely folded) bent state, and reversing back). As shown in Figure 6e, CE was decreased by less than 1% upon the folding test, and it recovered to its original value after unfolding. The high flexibility of the CC electrode and the keen electrode/electrolyte contact driven by the viscous DES electrolyte contribute to the robust cyclability of the SPDZ. To demonstrate the potential use of the DES‐ZBB in consumer‐level products such as wearable devices, a bracelet‐type battery was fabricated by connecting two SPDZs in series and successfully lit a red‐light LED (working voltage: 2.3–3.3 V, Figure 6f). The flexible DES‐ZBB is highly scalable, as demonstrated by the large‐area SPDZ with an active area of 20 cm^2^ (Figure 6g). It delivered cycling performance similar to that of a small SPDZ (5.4 mAh cm^−2^ for both SPDZs’ AADC). The simple cell architecture and robust cell performance endowed by the BCM DES present the strong potential of the DES‐ZBB for use in a range of energy storage devices.

## 3 Conclusion

In this work, we designed a DES electrolyte by simply mixing ZnBr_2_, ZnCl_2_, and MEPBr and demonstrated a DES‐based ZBB. By virtue of the water‐free feature and superior Br_2_ adsorption ability of the electrolyte, the DES‐ZBB could be operated in a closed coin‐type configuration without any gas generation. Experimental and computational analyses elucidated the reconstruction of MEPBr and the formation of a Zn‐halide complex anion. The inclusion of Cl^−^ not only affected the ion mobility but also improved the cycling stability and reaction kinetics, resulting in better cycling performance of the DES‐ZBB with Cl^−^. By exploiting the gas‐evolution‐free feature of the BCM DES, a flexible pouch‐type DES‐ZBB was successfully demonstrated. Considering the burgeoning interest in DESs, the novel DES‐ZBB presented here provides new insights into the development of DES‐based dual‐ion battery systems.

## 4 Experimental Section

### Preparation of DES

Each DES was prepared by simply mixing the required amounts of ZnBr_2_, ZnCl_2_, and MEPBr at 115 °C without any further treatment. In order to dissolve the parent salts completely, the DESs were kept in an oven for more than three days before use. Final products were in the form of transparent brown liquids.

### Characterization

The thermal properties of the prepared DESs were analyzed by means of ultralow temperature differential scanning calorimetry (DSC 214 Polyma, NETZSCH) with scan rates of 10 °C min^−1^ in a mixed gas condition (air/N_2_ = 4). The viscosity of each DES was measured with a RM 100 CP2000 PLUS viscometer (Lamy Rheology). Every measurement was conducted with a shear rate of 5 s^−1^ at 25 °C. X‐ray photoelectron spectroscopy (XPS; K‐alpha, Thermo VG Scientific) was used to investigate the core‐level electron states of the samples. The UV–vis spectrum of the charged DES electrolyte was ascertained with GENESYS 10s. For the analysis, the electrolytes were diluted with pure H_2_O. The Raman spectra were collected using an ARAMIS dispersive Raman spectrometer (Horiba Jobin Yvon) with an excitation laser wavelength of 514 nm. Zn deposition morphologies were characterized by scanning electron microscopy (SEM; Sirion, FEI Company). Solid‐state ^13^C NMR analyses were conducted using a Bruker AVANCE II 400 MHz spectrometer with a 9.4 T magnetic field. Hydrogen evolution was analyzed using an Agilent Technologies 7890A gas chromatography system.

### Cell Preparation and Electrochemical Measurements

DES‐based zinc bromine battery cell tests were conducted using CR2032 coin‐type cells. The coin cells were fabricated in an air condition using 15 pi Zn metal (25 µm) as the negative electrode, 12 pi carbon cloth as the positive electrode, and a DES‐soaked glass fiber separator. For the anode‐less system, the 15 pi carbon cloth was used as a negative electrode instead of Zn metal. The carbon cloth was heat‐treated at 520 °C for nine hours in air before use. Symmetric cells were prepared by replacing the 12 pi carbon cloth with 12 pi Zn metal. Electrochemical impedance spectroscopy (EIS) spectra were collected using a BioLogic VSP electrochemical workstation in a frequency range of 0.1–10^6^ Hz. Every galvanostatic charge‐discharge (GCD) test using the coin‐type cells was performed in a temperature maintainer (temperature fixed at 25 °C) using a TOSCAT‐3000U device (Toyo System). Regarding the full cell test, the current density and areal capacity conditions of the charge processes are included in the manuscript for each test. The discharge process also used the same current density with a charge process, though it was terminated with a 0 V voltage cut‐off condition. The symmetric cell tests were performed at 0.1 mA cm^−2^/0.05 mAh cm^−2^. The configuration of the three‐electrode cell is provided in Figure 4a. In order to observe the voltage profiles and EIS spectra of the redox reactions at the negative and positive electrode separately, Zn metal was used as a pseudo reference electrode. All electrochemical tests using the three‐electrode system were conducted with a VSP workstation.

### Computational Methods

Every calculation was performed with the Material Studio (BIOVIA, 2020) software package. The coordination structures of the Zn ions and halide anions were investigated by using the Forcite module for molecular dynamics (MD) calculations. Geometric optimizations were initially conducted with the COMPASS III force field at an ultrafine quality (2 × 10^−5^ kcal mol^−1^ for the convergence tolerance of energy, 1 × 10^−3^ kcal mol^−1^ Å^−1^ for the force, and 1 × 10^−4^ Å for the displacement). After geometric stabilization of the system, sequential NPT (constant number, pressure and temperature) ensembles and NVT (constant number, volume and temperature) ensembles were used to ensure equilibrium of the system for 2 ns each at 298 K. The system was stabilized further using 5 ns of NVT ensembles at 298 K, and the radial distribution function (RDF) spectra were collected. The binding energy calculations were carried out using the DMol^3^ module for DFT calculations in the same software. The Perdew–Burke–Ernzerhof generalized gradient approximation (GGA‐PBE) functional was used to express the electron exchange correlation, and spin polarization was unrestricted in all calculations. Double numerical plus polarization (DNP+) was used as the basis set. For the DFT calculations, the convergence tolerances of the energy, maximum force, and maximum displacement were set to 1 × 10^−7^ Ha, 2 × 10^−3^ Ha Å^−1^, and 5 × 10^−3^ Å, respectively.

## Conflict of Interest

The authors declare no conflict of interest.

## Supporting information

Supporting Information

## Data Availability

The data that support the findings of this study are available from the corresponding author upon reasonable request.
