# Supplementary material for: A Zinc–Bromine Battery with Deep Eutectic Electrolytes
Source: Adv Sci (Weinh). 2022 Oct 30;9(36):2204908. doi: 10.1002/advs.202204908 (PMC9798974; doi:10.1002/advs.202204908)
Supplement: Supplementary file 1 — Supporting Information [file ADVS-9-2204908-s001.pdf]

ADVANCED SCIENCE
Open Access

Supporting Information

for *Adv. Sci.*, DOI 10.1002/advs.202204908

A Zinc–Bromine Battery with Deep Eutectic Electrolytes

*Jiyun Heo, Kyungjae Shin and Hee-Tak Kim**

Supporting Information

A zinc-bromine battery with deep eutectic electrolytes

*Jiyun Heo, Kyungjae Shin, and Hee-Tak Kim **

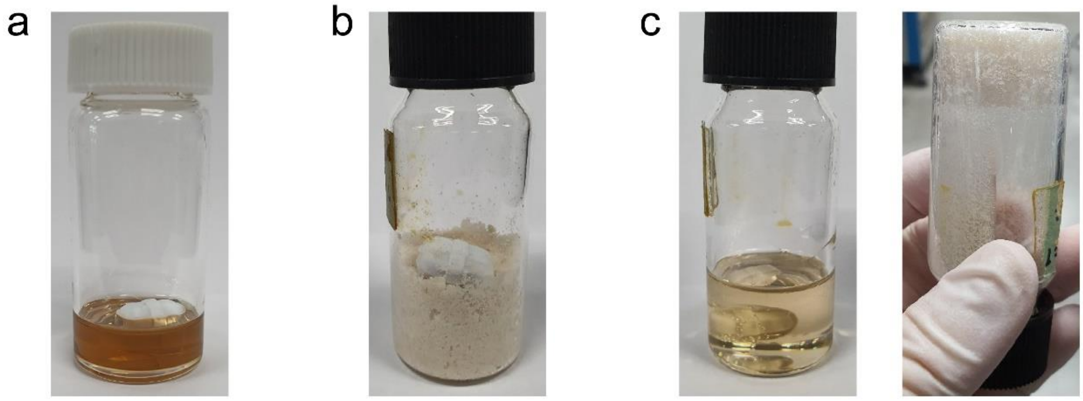

Figure S1. DES preparation with various BCAs. a) MEPBr-based DES at room temperature, b) TPABr-based mixture at 115 °C, and c) TBABr-based DES at 115 °C (left) and at room temperature (right)

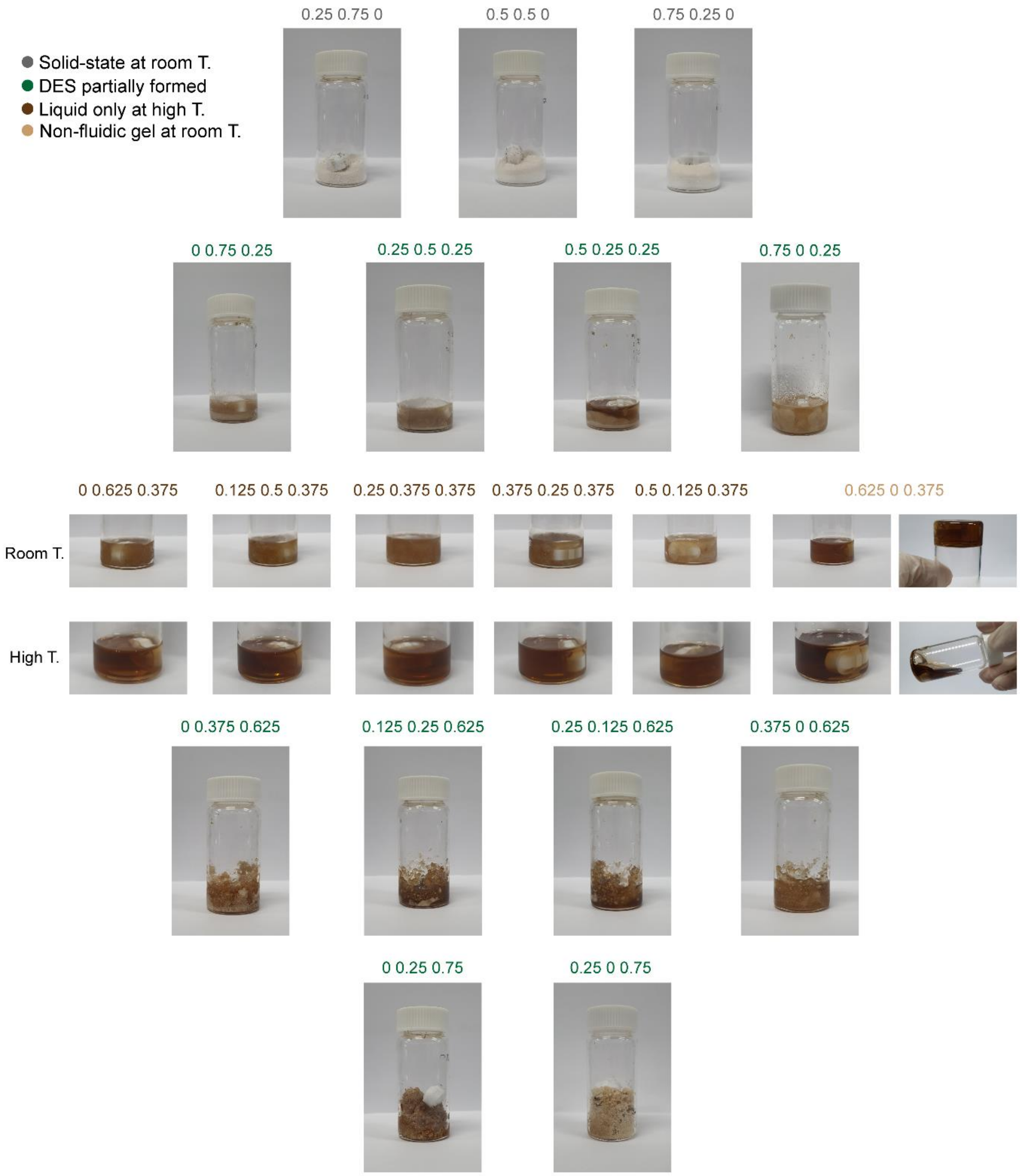

Figure S2. DES preparation at various ZB : ZC : MEPBr ratios

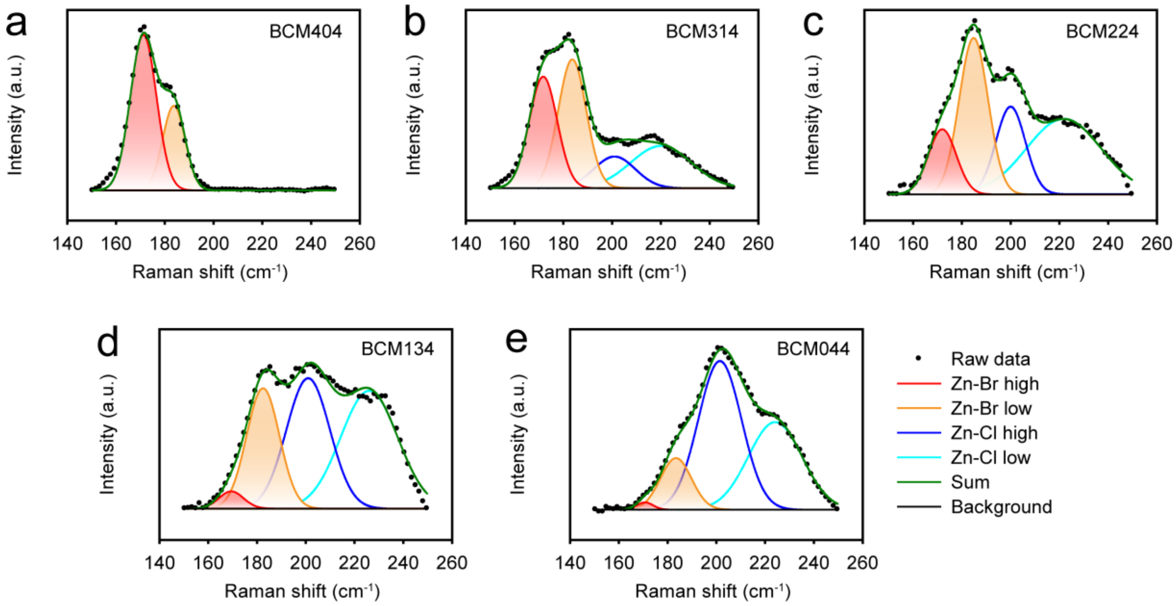

Figure S3. Deconvoluted Raman spectra of BCM a) 404, b) 314, c) 224, d) 134, and e) 044 in the range of 150 to 250 cm^{-1}, where Zn-halide stretching modes appear. The Zn-Br or Zn-Cl peaks were deconvoluted into two peaks depending on the halide coordination number (high coordination: >2, low coordination: ≤ 2).

Raman signals of Zn-halide bonds were deconvoluted according to the coordination number as shown in Figure S3.[1–5] With the increase of the Cl^-/Br^- ratio, the peaks of the Zn-Br bond decreased and that of the Zn-Cl bond increased. The presence of the Zn-Br peaks for BCM044 indicates the reconstruction process of DES.

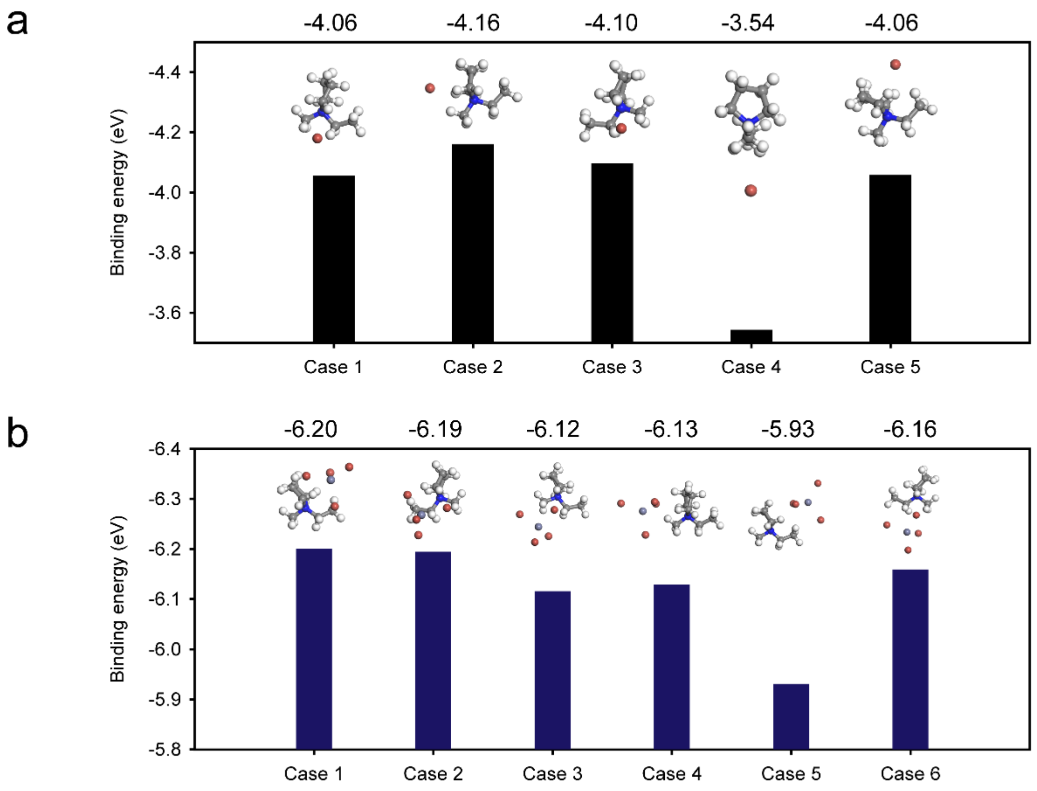

Figure S4. Binding energy between MEP^+ and the counter anion (Br^- for powder MEPBr and $[ZnBr_4]^{2-}$ for the DES electrolyte) from the DFT calculation. a) Powder MEPBr, b) DES electrolyte

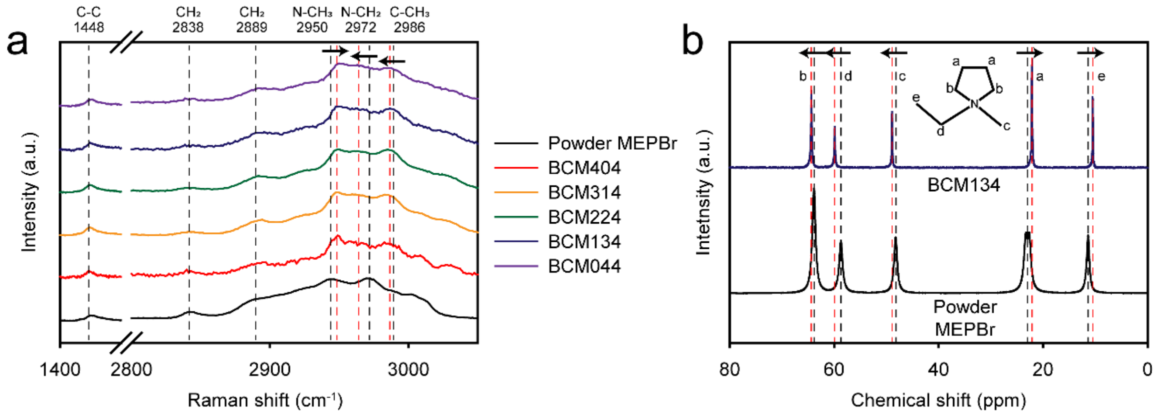

Figure S5. MEPBr binding conformation analyses. a) Raman spectra of powder MEPBr and BCM 404 ~ 044, and b) solid-state ^{13}C NMR spectra of powder MEPBr and BCM134

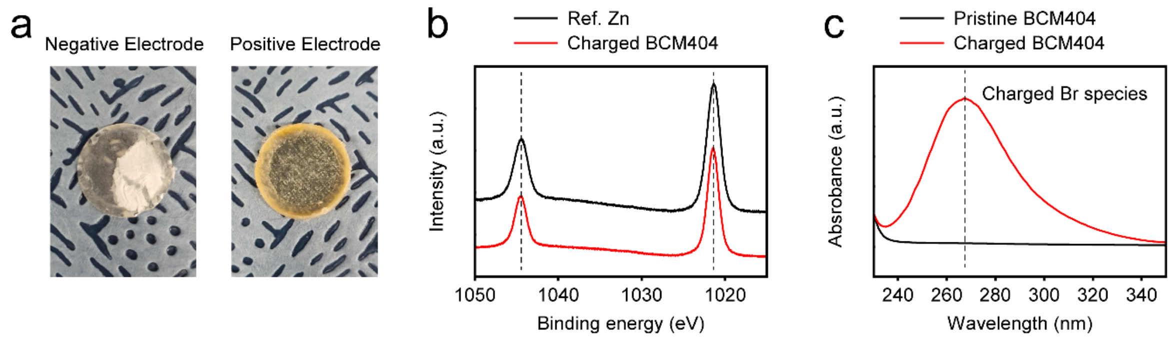

Figure S6. Post-mortem analyses of charged DES-ZBB with BCM404. a) Optical images of Zn deposited on a negative electrode with entangled glass fibers (left) and charged Br species indicated by the characteristic yellow color at the separator facing the positive electrode (right), b) XPS spectrum of Zn 2p detected on a glass fiber separator on the negative electrode side, and c) UV-Vis spectrum of the charged BCM404 electrolyte in the positive electrode compared to pristine BCM404 diluted with H_2O

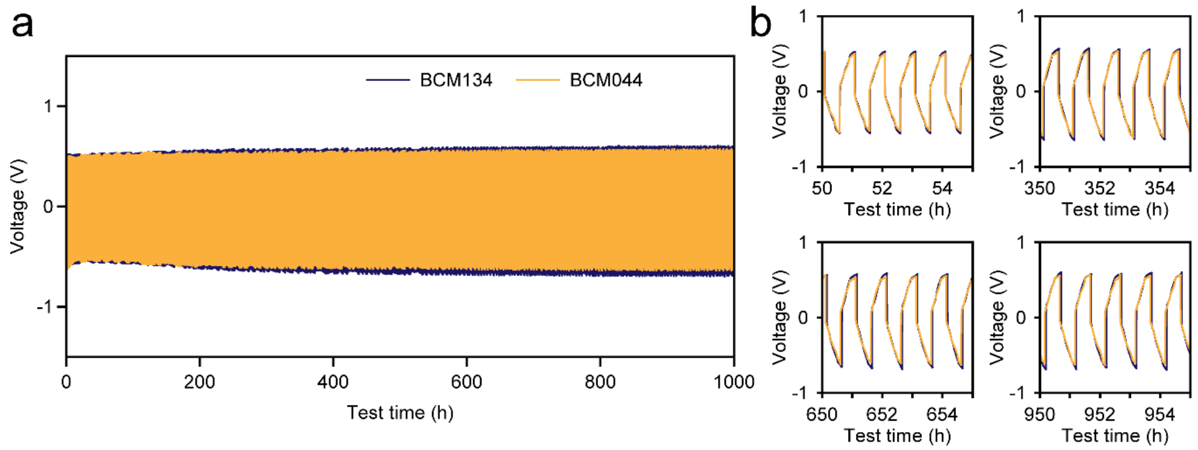

Figure S7. Long-term galvanostatic cycling test using a symmetric Zn/Zn cell with BCM134 and BCM044. a) full-range voltage profiles; and b) voltage profiles at 50 h, 350 h, 650 h, and 950 h

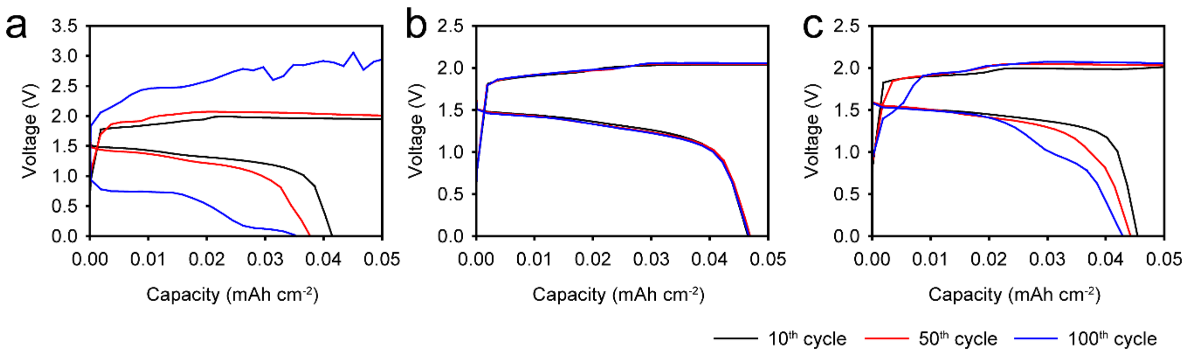

Figure S8. Voltage profiles of DESs at 10th, 50th, and 100th cycle examined under the 0.1 mA cm^{-2}/ 0.05 mAh cm^{-2} conditions. a) BCM224, b) BCM134, and c) BCM044

As shown in Figure S8, the voltage profiles of the BCMs show differences according to the Cl^- anion ratio. For the case with the low Cl^- ratio (BCM224), the overpotentials of both the charge and discharge processes increase during the cycles. This occurs because the charged Br species could not be fully reduced during the discharge process but instead accumulated, reducing the ionic conductivity and increasing the overpotential. On the other hand, when the Cl^- ratio is too high (BCM044), the self-discharge of charged Br species can be accelerated, consuming Zn metal and impinging on the discharge process. Therefore, the optimized Cl^- anion ratio (BCM134) is required to maximize the cycling performance of DES-ZBB.

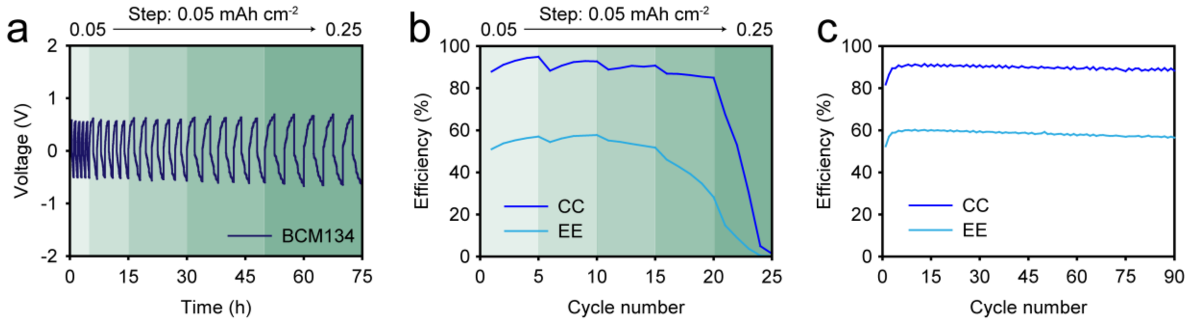

Figure S9. Areal capacity capability test using BCM134 DES-ZBB. a) Symmetric Zn/Zn cell test, and b) Zn/CC full cell test at fixed current density (0.1 mA cm^{-2}) with varying areal capacity from 0.05 mAh cm^{-2} to 0.25 mAh cm^{-2}. c) Long-term full cell test conducted under 0.1 mA cm^{-2} / 0.1 mAh cm^{-2} test condition

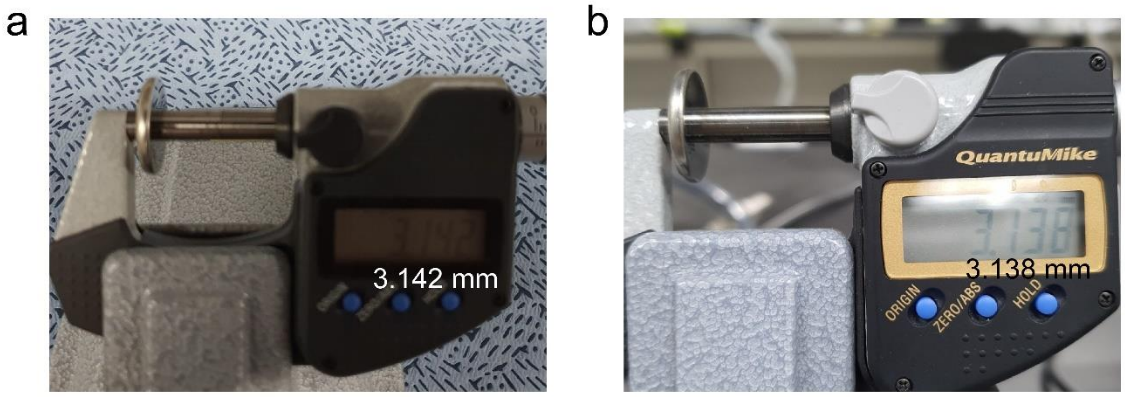

Figure S10. Optical image of the cell thickness a) before and b) after the cycling test

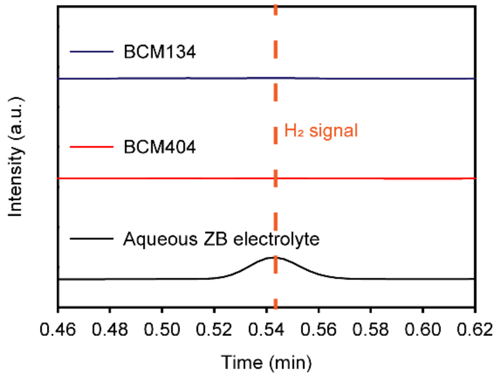

Figure S11. H_2 gas chromatography test after the immersion of the Zn metal in each electrolyte for one week

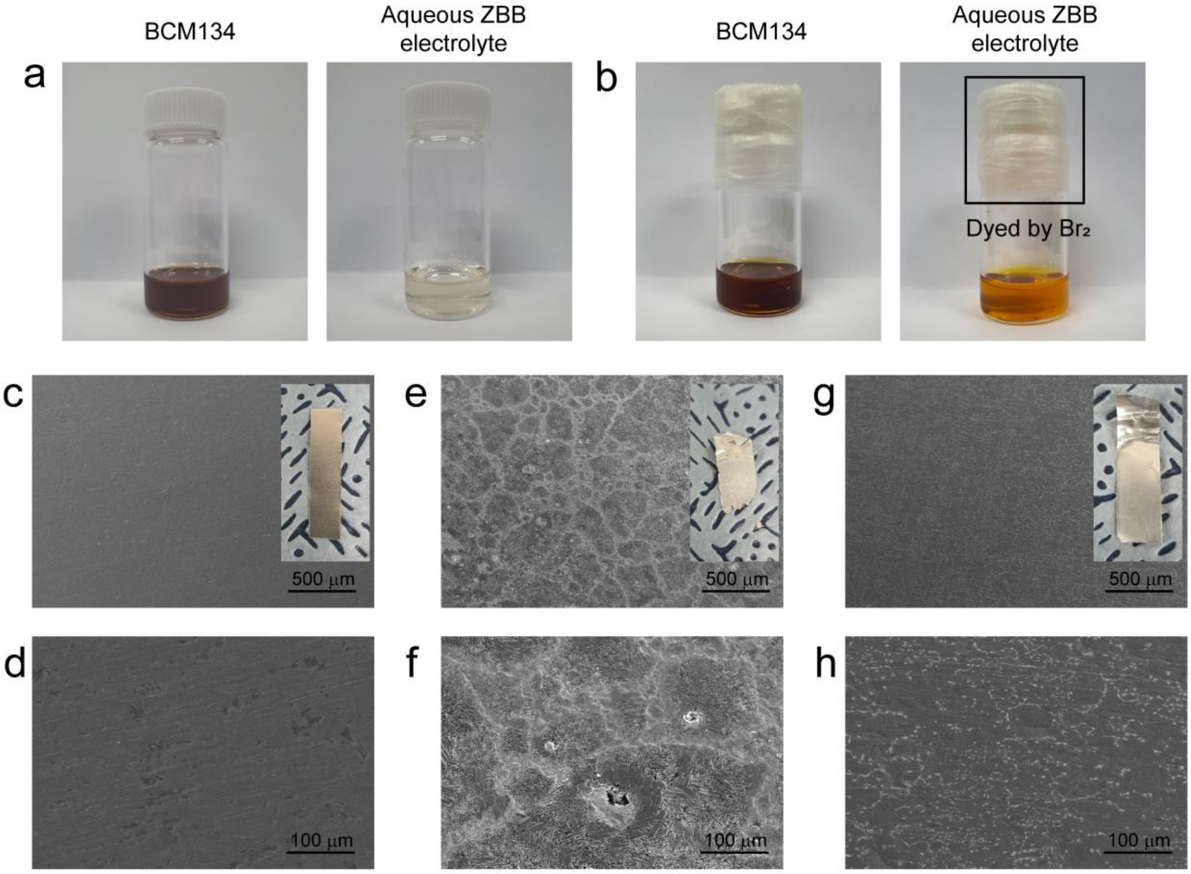

Figure S12. Br_2 evaporation test. a) Pristine BCM134 and aqueous ZBB electrolyte (2 M ZB + 0.5 M MEPBr), b) each electrolyte 12 h after the addition of liquid Br_2. SEM image of c), d) pristine Zn metal, Zn metal stored for 12 h in contact with the vapor equilibrated with e), f) Br_2-containing aqueous ZBB electrolyte (2 M ZB + 0.5 M MEPBr), and g), h) Br_2-containing BCM134 electrolyte. Inset images in c), e), and g) are the digital images of Zn metals analyzed in each SEM image.

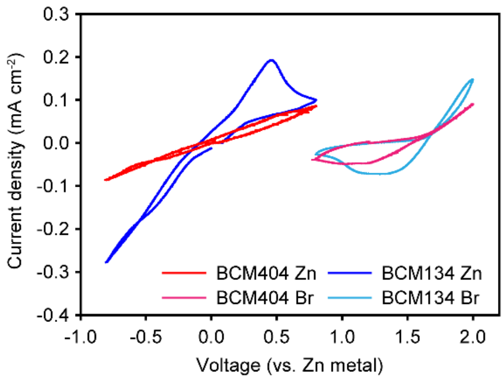

Figure S13. Cyclic voltammetry test of BCM404 and BCM134 using a three-electrode cell (scan rate: 1 mV s^{-1})

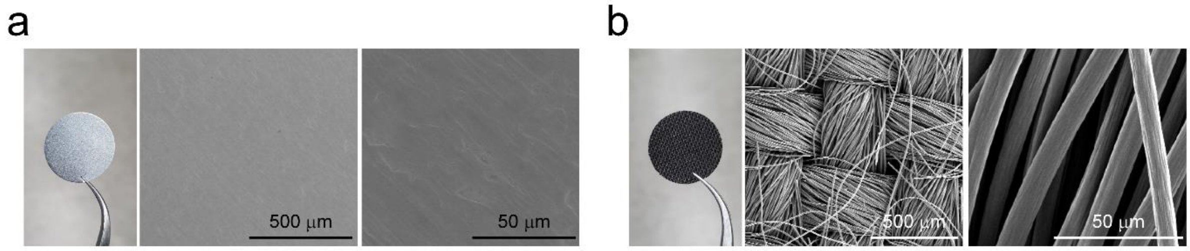

Figure S14. Optical and SEM images of pristine a) Zn metal and b) carbon cloth electrodes

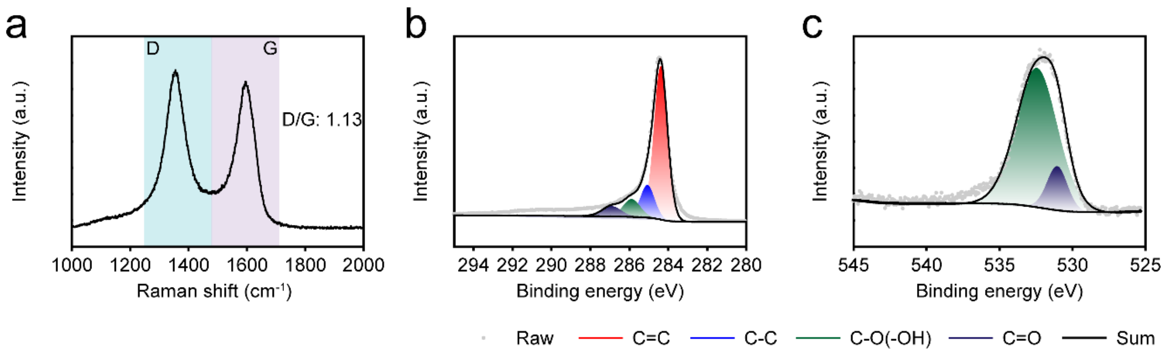

Figure S15. Chemical structural analyses of the carbon cloth electrode. a) Raman spectrum, and XPS spectra of b) C 1s and c) O 1s

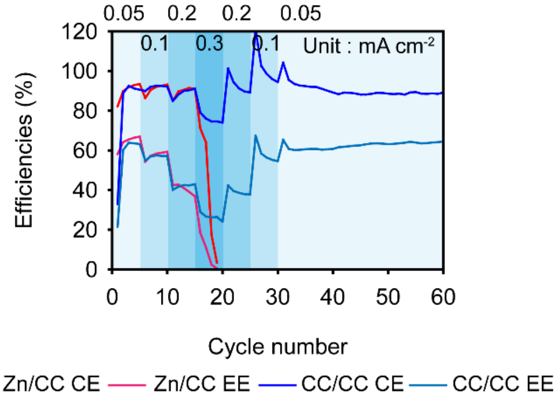

Figure S16. Rate capability test with varying current densities from 0.05 to 0.3 mA cm^{-2} with a fixed charging time of 30 min

Table S1. Concentrations of each ion and thermodynamic redox potentials of Br^-/Br_2 versus Zn^{2+}/Zn in BCM404 and BCM134

Sample	Density (g mL^{-1})	Specific Br density (g mL^{-1})	Br concentration (mol L^{-1})	Specific Zn density (g mL^{-1})	Zn concentration (mol L^{-1})	Redox potential (V)
BCM404	2.014	1.153	14.43	0.316	4.83	1.74
BCM134	1.741	0.591	7.40	0.325	4.96	1.76

The thermodynamic redox potential of Br^-/Br_2 was calculated using the Nernst equation, as expressed below,

$$E = E^\circ - \frac{RT}{2F}\ln([Zn^{2+}] \times [Br^-]^2),$$

where E° is the standard redox potential difference between Br^-/Br_2 (1.066 V vs. SHE) and Zn^{2+}/Zn (-0.762 V vs. SHE), R is the ideal gas constant, T is 298 K, and F is Faraday constant. Considering the specific densities of Br^- anions and Zn^{2+} cations in each DES, the Br^- concentration and Zn^{2+} concentration can be obtained, as shown in Table S1. Using these values, the thermodynamic redox potential of Br^-/Br_2 versus Zn^{2+}/Zn can be calculated as 1.74 V and 1.76 V for BCM404 and BCM134, respectively.

Table S2. Qualitative comparison of the resistance values at negative and positive electrodes using BCM404 and BCM134

Unit: Ω	Zn^{2+}/Zn R_{ct}	Film resistance from the complexing layer	Br_2/Br^- R_{ct}
BCM404	12021	385	1621
BCM134	2854	336	511

Supplementary Note S1. Additional explanation of the role of Cl^- on the discharge overpotential

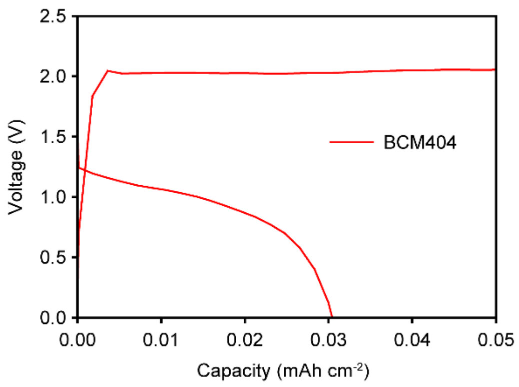

Figure S17. Voltage profile of BCM404 using anode-less cell. The test was conducted under 0.1 mA cm^{-2} / 0.05 mAh cm^{-2} condition.

In Figure 3a, there are distinguishable discharge stages for the BCMs with low Cl^- content (BCM404, and 314). Based on the large overpotential of the negative electrode, we believe that this behavior is attributed to the different overpotentials between the dissolution of deposited Zn and the formation of pits on the Zn metal electrode. It is commonly accepted that there is an abrupt increase in overpotential for the pit-formation process in metallic anodes.[6,7] This assumption could be verified by the absence of the lower voltage region when a carbon cloth electrode was used for BCM404 (Figure S17). Moreover, we could also notice an increment in overpotential at the late stage in each cycle from our symmetric cell test, and the increment is larger with low Cl^- containing BCMs. This indicates that the different dissolution reaction in the Zn metal electrode is the origin of the two-stage discharge process.

References

[1] T. Takamuku, K. Yoshikai, T. Yamaguchi, H. Wakita, *Zeitschrift für Naturforsch. A* **1992**, *47*, 841.
[2] J. Van Heumen, T. Ozeki, D. E. Irish, *Can. J. Chem.* **1989**, *67*, 2030.
[3] K. Kinugawa, K. Kadono, H. Tanaka, *J. Non. Cryst. Solids* **1989**, *110*, 265.
[4] R. M. Almeida, *J. Non. Cryst. Solids* **1987**, *95–96*, 279.
[5] O. G. Parchment, M. A. Vincent, I. H. Hillier, *J. Phys. Chem.* **1996**, *100*, 9689.
[6] K. N. Wood, E. Kazyak, A. F. Chadwick, K. H. Chen, J. G. Zhang, K. Thornton, N. P. Dasgupta, *ACS Cent. Sci.* **2016**, *2*, 790.
[7] J. Heo, Y. Hwang, G. Doo, J. Jung, K. Shin, D. Koh, H. Kim, *Small* **2022**, *18*, 2201163.
